# Early feeding of larger volumes of formula milk is associated with greater body weight or overweight in later infancy

**DOI:** 10.1186/s12937-018-0322-5

**Published:** 2018-01-24

**Authors:** Junmei Huang, Zhen Zhang, Yuanjue Wu, Yan Wang, Jing Wang, Li Zhou, Zemin Ni, Liping Hao, Nianhong Yang, Xuefeng Yang

**Affiliations:** 1 Department of Nutrition and Food Hygiene, Hubei Key Laboratory of Food Nutrition and Safety, School of Public Health, Tongji Medical College, Huazhong University of Science and Technology, 13 Hangkong Road, Wuhan, Hubei 430030 People’s Republic of China; 2 Jiangan Maternal and Child Health Hospital, Wuhan, Hubei 430014 China; 3 Jiangan Centers for Disease Control and Prevention, Wuhan, Hubei 430014 China

**Keywords:** Feeding practice, Formula milk, Growth, Overweight, Greater body weight

## Abstract

**Background:**

The relation between infant feeding and growth has been extensively evaluated, but studies examining the volume of formula milk consumption on infant growth are limited. This study aimed to examine the effects of early feeding of larger volumes of formula on growth and risk of overweight in later infancy.

**Methods:**

In total, 1093 infants were studied prospectively. Milk records collected at 3 mo of age were used to define the following 3 feeding groups: breast milk feeding (BM, no formula), lower-volume formula milk feeding (LFM, <840 ml formula/d), and higher-volume formula milk feeding (HFM, ≥840 ml formula/d). Body weight and length were measured at 3 time points of 3, 6 and 12 mo of age.

**Results:**

The results showed that the difference in weight and length between the HFM and BM infants was significant at 3 mo of age (*P* < 0.05) and continued until 12 mo of age (*P* < 0.001). The adjusted mean changes in weight-for-length z-scores (WLZ) and BMI-for-age z-scores (BAZ) from 3 to 6 mo of age were significantly higher in HFM and LFM group than in BM group. Two-way interactions between feeding practice and age intervals were significant for WLZ changes (*P* = 0.002) and BAZ changes (*P* = 0.017). Compared with BM-fed infants, infants fed with HFM had 1.60-fold (95% CI 1.05–2.44) higher odds of greater body weight (1SD < WLZ ≤2 SD) at the age of 6 mo and 1.55-fold (95% CI 1.01–2.37) higher odds of greater body weight and 2.13-fold (95% CI 1.03–4.38) higher odds of overweight (WLZ > 2 SD) at the age of 12 mo.

**Conclusion:**

Feeding higher volumes of formula in early infancy is associated with greater body weight and overweight in later infancy.

## Background

The prevalence of overweight and obesity in children is a major health problem worldwide. Early infancy is a period of fast growth and weight gain, and infants who gain more weight during infancy become susceptible to obesity in childhood or later life [1–5]. Feeding patterns such as breastfeeding or formulas-feeding are the main factors that affect the growth and development in infancy [6, 7]. Some studies have primarily focused on the association between various feeding patterns in infancy and the risk of overweight and obesity in childhood and adulthood. It is generally acknowledged that formula-fed infants gain more weight than breastfed infants and breastfeeding is an important protector against overweight and obesity [8–12]. However, few studies have focused on differences in the growth and risk of overweight in infants fed with different type and volume of milk in early infancy.

The difference in weight gain between formula-fed and breast-fed infants is likely to be related to differences in both the composition and volume of intake between formula and breast milk. A systematic review concluded that formula-fed infants have a 1.2-to 9.5-fold higher energy intake and a 1.2-to 4.8-fold higher protein intake than those who are breastfed in the first week of life [13]. This difference is attributed to the higher energy and protein content of formula and a higher volume of consumption, which may lead to greater weight gain in formula-fed infants compared to breastfed infants during early infancy [13]. This review suggested that higher amounts of formula consumption may expose formula-fed infants to energy-dense milk, leading to a greater risk of overweight. Hopkin’s study examined the effect of the type and volume of milk fed during infancy on childhood growth, and the results showed that feeding infants high volumes of formula (≥600 ml/d) was associated with increased body weight and height through 3 y of age and suggested that milk intake should be measured in detail in future research [14]. Another study observed the relationship between bottle size and weight gain in formula-fed infants and found that using a large bottle in early infancy independently contributed to greater weight gain and change in weight-for-length z-scores (WLZ) at the age of 6 mo [15]. Therefore, feeding larger volumes of formula may be associated with greater body weight and length gains.

The infant formula has been widely used in China over the last decade and about 65.3% infants aged within 6 mo has consumed infant formula [16]. However,whether the large amount of formula intake in early infancy may contribute to the increased prevalence of overweight and obesity in later life has not been extensively studied. Thus, the present study aimed to investigate the association between the volume of formula milk consumption at 3 mo of age and changes in body weight and length at 3 time points during the first year, and to examine the effects of different volumes of formula milk during early infancy on growth and risk of overweight in later infancy.

## Methods

### Study design

The data were collected from a subgroup of the Tongji Maternal and Child Health Cohort (TMCHC) study, which was described previously [17]. Using a population-based, prospective, observational study design, TMCHC collected detailed information on pregnant women and their infants and to investigate the influence of dietary factors on maternal and child health during pregnancy and infancy. This study was approved by the Institutional Review Board of the Tongji Medical College of Huazhong University of Science and Technology and therefore was performed in accordance with the ethical standards outlined in the Declaration of Helsinki. The written informed consent was obtained from participants’ families before participants were included in the study.

Healthy neonates (*n* = 1229) who were born between March 2014 and June 2015 from TMCHC and who were followed up with until 1 year of age were screened for enrollment in the present study. Neonates with birth defects or congenital long-term diseases were excluded.. The sample size of 1093 was estimated using the simple random sampling method ($n={\left({Z}_{\alpha /2}\right)}^2\times \frac{1}{\varepsilon^2}\times \frac{1-p}{p}\alpha =0.05,\upvarepsilon =0.22$), and we used 6.7% as the estimated rate of overweight and obesity at 1 year of age (*P* = 6.7%) [18]. We used 11% as the estimated rate of lost-to-follow up, the sample size at recruitment was 1093/(1–0.11) = 1229.

Information about maternal characteristics such as age, education, pre-pregnancy height and weight, occupation, gravidity, parity, health condition and delivery, and infant characteristics including sex, gestational age, mode of delivery and birth weight and length were obtained by the Maternal and Children Healthcare Information Tracking System of Wuhan. After enrollment, the participants were interviewed by a community physician 3 times: at 3 months, 6 months and 12 months postpartum. At each of these interviews, data on infant feeding practice, sleep duration, illness, dietary supplement intake, introduction of solid foods were obtained. Body weight and length was measured by the trained community physician. Body weight was measured to the nearest 100 g using a pedobarometer, with infants wearing light indoor clothing. Recumbent length was measured to the nearest 0.1 cm using an infantometer. Both weight and length were taken in duplicate and means of the replicates were recorded. Detailed records of formula consumption were collected at the age of 3 mo.

**Fig. 1:**
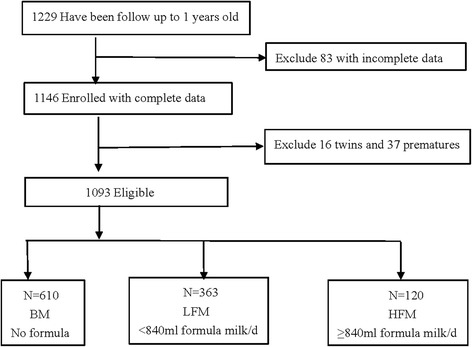
Enrollment, eligibility and study sample. Feeding patterns at the age of 3 mo: BM, no formula milk; LFM, <840 ml formula milk/d; HFM, ≥840 ml formula milk/d

In total, 1146 infants completed examinations at all growth points. After excluding twins (*n* = 16) and premature infants (*n* = 37),1093 healthy singleton full-term infants were ultimately selected for analysis, including 587 boys and 512 girls (Fig. 1 and Table 1).

**Table 1:** Differences between maternal and infant characteristics and volume of formula milk consumption at 3 mo of age^a^

	Feeding groups				
	Overall	BM	LFM	HFM	*P*
Maternal characteristics		610(55.5)	363(33.2)	120(11.0)	
Age(years), n(%)					
17~25	134(12.4)	83(13.6)	42(11.6)	9(7.5)	0.022
25~29	562(51.8)	319(52.3)	192(52.9)	51(42.5)	
30~34	305(28.1)	156(25.6)	99(27.3)	50(41.7)	
35~46	83(7.7)	45(7.4)	28(7.7)	10(8.3)	
Educational level(years), n(%)					
≤ 9	139(12.8)	83(13.6)	41(11.3)	15(12.5)	0.657
10~12	248(22.9)	129(21.1)	90(24.8)	29(24.3)	
13~22	695(64.2)	390(63.9)	229(63.1)	76(63.3)	
BMI before pregnancy(kg/m^2^), n(%)					
14.42~18.5	233(21.4)	121(19.8)	84(23.1)	28(23.3)	0.022
18.5~23.9	722(66.2)	416(68.2)	238(65.6)	68(56.7)	
24~27.9	115(10.6)	65(10.7)	29(8.0)	21(17.5)	
28~31.25	20(1.8)	7(1.1)	10(2.8)	3(2.5)	
Maternal gestational weight gain quartile (kg), n(%)					
2~13	275(25.3)	154(25.2)	84(23.1)	37(30.8)	0.132
13.1~16.5	270(24.8)	156(25.6)	97(26.7)	17(14.2)	
16.6~20	307(28.2)	171(28.0)	97(26.7)	39(32.5)	
20.1~41	237(21.8)	127(20.8)	83(22.9)	27(22.5)	
Cesarean, n(%)					
yes	680(62.8)	352(57.7)	231(63.6)	97(80.8)	<0.001
Maternal GDM, n(%)					
yes	60(5.5)	37(6.1)	15(4.1)	8(6.7)	0.501
Return to work at postpartum 3 month, n(%)					
yes	121(11.1)	39(6.4)	57(15.7)	25(20.8)	<0.001
Infant characteristics					
Sex, n(%)					
Boy	585(53.5)	302(49.5)	209(57.6)	74(61.7)	0.008
Girl	508(46.5)	308(50.5)	154(42.4)	46(38.3)	
Infant gestational age at birth(wk), n(%)					
37~38	306(28.2)	157(25.7)	115(31.7)	34(28.3)	0.226
39~40	700(64.6)	407(66.7)	217(60.0)	76(63.3)	
41~42	78(7.2)	39(6.4)	29(8.0)	10(8.3)	
Age at introduction of solid foods(months), n(%)					
< 6	680(63.7)	355(58.2)	238(65.6)	87(72.5)	0.013
≥ 6	387(36.3)	233(38.2)	123(33.9)	31(25.8)	
illness episodes, n(%)					
yes	137(12.5)	72(11.8)	51(14.0)	14(11.7)	0.566
Daily sleep time at 3mo(h)	14.96 ± 1.97^b^	15.04 ± 1.9	14.83 ± 2.04	14.95 ± 2.1	0.282
Daily sleep time at 6mo(h)	13.63 ± 1.48	13.66 ± 1.51	13.56 ± 1.47	13.71 ± 1.41	0.525
Formula volume intake (ml/d) P50(P10; P90)	600(120 ;960)	0	405(120;720)	960(840;1120)	

^a^Actual *n* = 1093. *P* values were determined on the basis of Pearson’s chi-square test of an association for categorical variables and the one-way ANOVA for continuous outcome variables. BM: breast milk, LFM:<840 ml formula milk/d; HFM: ≥840 ml formula milk/d.^b^ Mean ± SD (all such values)

### Infant feeding assessments

Infant feeding patterns were categorized according to the total volume of formula milk consumed at the age of 3 mo, which was calculated by caregivers’ answers to 2 questions: “How many times was your child fed infant formula?” and “How much formula milk does your child usually intake at each feeding?” The caregivers was also inquired about how to prepare the formula solution for infants. Generally, the caregivers prepared the formula milk according to the instruction of manufacturers by using measurements provided by manufacturers. To verify the volume of formula consumption provided by the caregivers, we compared the volume of formula consumption recorded by community physician at their home visit with the intake reported by the caregivers in randomly selected 100 formula-fed infants at the age of 3 mo in the present study. The quantities are comparable in two independent reports recorded by community physician and the caregivers for 100 infants. Based on Chinese infant feeding recommendations and references to the dietary intake of Chinese residents [19, 20], we divided the volume of formula into higher (840 ml or more) and lower (less than 840 ml) consumption as the 3-month-old infants consumed an average of 140 ml of formula 6 times/d for a total of 840 ml/d, which was P75 of formula intake in formula-fed infants in the present study. The 3 feeding groups were identified as follows: breast milk feeding (BM, no formula milk); lower-volume formula milk feeding (LFM, <840 ml formula milk/d); and higher-volume formula milk feeding (HFM, ≥840 ml formula milk/d). Breast milk feeding was defined as infants who exclusively consumed breast milk without other liquids or solids with the exception of vitamin and mineral supplements or medicines. Formula feeding included any formula feeding with or without some breast milk and was subdivided into LFM and HFM according to the volume of formula milk intake per day.

### Anthropometric indexes

For our analysis, we calculated age-and sex-specific z scores for the following 2 anthropometric indexes: weight-for-length z score (WLZ) and BMI-for-age z score (BAZ) using WHO Anthro software (version 3.2.2) based on the 2006 WHO growth standards [21]. To assess longitudinal growth, interval growth changes between 3 feeding groups were determined by obtaining the differences in WLZ and BAZ from birth to 3 mo, 3 to 6 mo, and 6 to 12 mo of age. The infants’ weight status was measured by WLZ and was classified as greater body weight (1SD < WLZ ≤ 2SD), which represented at-risk for overweight, and overweight (WLZ > 2SD).

### Statistical analyses

Epidata3.1 database software was used for double entry and validation. Chi-square tests for categorical variables and ANOVA for continuous variables were performed to explore differences in maternal and infant characteristics between feeding groups. All data including weight, length, WLZ and BAZ were expressed as the mean ± standard deviation (x ± s) and analyzed using a one-way ANOVA to compare differences between feeding groups (LFM and HFM versus BM to understand the impact of formula milk exposure).

To test the effect of formula consumption on changes in WLZ and BAZ, linear mixed-effect modeling was used to analyze repeated measurements. Adjusted confounders including infant sex, infant birth weight, cesarean delivery, pre-pregnancy BMI and weight gain during pregnancy, and WLZ or BAZ at birth, which have previously been shown to influence infant growth, were centered at their mean values in the multivariate model. The basic model included feeding groups (BM, LFM or HFM) and age intervals (0, 3, 6 and 12 mo of age) and interactions between feeding practice and age intervals. In addition to these fixed effects, a random effect that represented the between-infant variability was included in the model.

Associations between formula consumption at 3 mo of age and infants’ weight status were also investigated using logistic regression analyses after adjusting for infant sex, infant birth WLZ, infant birth weight, introduction of solid foods, cesarean delivery, pre-pregnancy maternal body mass index and weight gain during pregnancy. All *P* values were 2-tailed, with *P* < 0.05 considered to be statistically significant. All analyses were performed using SPSS software (IBM SPSS Statistics V21.0.).

## Results

### Descriptive

For this study,1093 healthy full-term infants were included in the final analysis. Based on their feeding practice at 3 months of age (Table 1), they were classified into the BM feeding group (610, 55.5%), LFM feeding group (363, 33.2%) and HFM feeding group (120, 13.2%). The results also showed that mothers who fed breast milk to their infants had a normal BMI before pregnancy (68.2% vs 65.6% vs 56.7%, *p* < 0.05) and no cesarean deliveries (42.3% vs 36.4% vs 19.2%, *p* < 0.001) compared to both LFM and HFM mothers. Overall, solid foods were introduced to 63.7% of infants before the age of 6 mo. With respect to the sex of the infants, 50.5% of the breastfed infants were female, more than that of LFM infants (42.4%) or HFM infants (38.3%) (*p* < 0.01) .

As shown in Table 2, there were no significant differences in birth weight and length between any of the feeding groups (*p* > 0.05). The average values of body weight and length at three time points were greater in infants in the HFM group than those in the BM groups(*p* < 0.05 or *p* < 0.01), but there was no significant difference between infants in the LFM and BM group except those at 12 mo (*p* < 0.001). The differences in WLZ between the HFM and BM infants were only significant at 12 mo of age (*P* < 0.001), the same as the differences between the LFM and BM infants (*p* < 0.01). The differences in BAZ were significant at both 6 (*p* < 0.05) and 12 mo (*p* < 0.01) of age between the HFM and BM infants, but only significant at 12 mo of age (*p* < 0.05) between the LFM and BM infants.

**Table 2:** Comparison of infants’ anthropometric indexes by volume of formula milk consumption at 3 mo of age

		Feeding groups						
Variable and age	n	BM	LFM	HFM	P^a^	P^b^	P^c^	P^d^
Body Weight (kg)		610(55.5)	363(33.2)	120(11.0)				
At birth	1093	3.33 ± 0.39	3.34 ± 0.38	3.36 ± 0.42	0.782	0.890	0.483	0.563
3mo	1085	6.93 ± 0.84	6.90 ± 0.79	7.12 ± 0.72	0.029	0.515	0.019	0.009
6mo	1093	8.51 ± 1.00	8.61 ± 1.02	8.85 ± 0.87	0.002	0.118	0.022	0.022
12mo	1093	10.11 ± 1.07	10.4 ± 1.10	10.73 ± 0.99	<0.001	<0.001	<0.001	0.004
Body Length (cm)								
At birth	1093	50.1 ± 1.24	50.28 ± 1.31	50.11 ± 1.28	0.095	0.035	0.963	0.199
3mo	1085	62.34 ± 2.16	62.5 ± 2.3	63.07 ± 2.47	0.005	0.275	0.001	0.016
6mo	1093	68.34 ± 2.33	68.64 ± 2.45	69.04 ± 2.51	0.007	0.057	0.004	0.117
12mo	1093	76.35 ± 2.41	76.92 ± 2.85	77.46 ± 2.61	<0.001	<0.001	<0.001	0.045
WLZ								
At birth	1093	−0.19 ± 1.03	−0.29 ± 0.99	−0.1 ± 1.00	0.147	0.143	0.369	0.076
3mo	1085	0.64 ± 1.08	0.51 ± 0.97	0.65 ± 0.8	0.150	0.064	0.198	0.198
6mo	1093	0.74 ± 1.02	0.76 ± 0.98	0.96 ± 0.93	0.080	0.749	0.055	0.055
12mo	1093	0.56 ± 0.93	0.73 ± 0.91	0.96 ± 0.81	<0.001	0.003	<0.001	0.020
BAZ								
At birth	1093	−0.15 ± 1.01	−0.21 ± 0.96	−0.08 ± 1	0.387	0.326	0.488	0.202
3mo	1085	0.68 ± 1.07	0.55 ± 0.95	0.7 ± 0.77	0.105	0.047	0.816	0.140
6mo	1093	0.64 ± 1.03	0.66 ± 1	0.86 ± 0.94	0.098	0.828	0.033	0.593
12mo	1093	0.5 ± 0.95	0.65 ± 0.93	0.83 ± 0.83	<0.001	0.012	<0.001	0.061

^a^One-way ANOVA for continuous variables were used to compare the 3 groups

^b^Comparison between BM and LFM.^c^ Comparison between BM and HFM.^d^ Comparison between LFM and HFM.BM, no formula milk; LFM, <840 ml formula milk/d; HFM,≥840 ml formula milk/d; WLZ, weight-for-length z-scores; BAZ, BMI-for-age z score

### Linear mixed-effect modeling results

The adjusted mean changes in WLZ and BAZ of infants are shown in Table 3. Overall, 2-way interactions between feeding practice and age intervals were significant for WLZ changes (*P* = 0.002) and BAZ changes (*P* = 0.017) after adjusting for potential confounders.

**Table 3:** Adjusted mean changes in WLZs and BAZs of infants^a^

Feeding groups/Age interval	WLZ change^b^ (*n* = 1054)	*P* values	BAZ change^c^ (*n* = 1054)	*P* values
BM,mo				
Birth─3	0.84(0.75,0.94)		0.84(0.75,0.93)	
3─6	0.12(0.05,0.20)		−0.01(−0.09,0.06)	
6─12	−0.19(−0.27,-0.12)		−0.16(−0.23,-0.08)	
LFM,mo				
Birth─3	0.79(0.66,0.91)	0.833	0.76(0.64,0.88)	0.753
3─6	0.26(0.16,0.35)*	0.016	0.12(0.03,0.21)*	0.013
6─12	−0.04(−0.13,0.06)*	0.018	−0.02(−0.12,0.08)	0.056
HFM,mo				
Birth─3	0.77(0.56,0.99)	0.833	0.80(0.60,1.00)	0.753
3─6	0.30(0.14,0.47)*	0.023	0.15(−0.01,0.31)*	0.033
6─12	0.00(−0.17,0.16)*	0.048	−0.02(−0.19,0.15)	0.056

^a^Values are means with 95% CIs in parentheses. Analyses were performed with the use of linear mixed-effect modeling and adjusted for infant sex, infant birth weight, cesarean, introduction of solid foods, illness episodes, pre-pregnancy BMI and weight gain during pregnancy, and WLZ or BAZ at birth. BM, no formula milk; LFM, <840 ml formula milk/d; HFM, ≥840 ml formula milk/d; WLZ, weight-for-length z-scores; BAZ, BMI-for-age z score. *Significantly different from BM at the same time interval determined by one-way ANOVA (*P* < 0.05). ^b^Significant 2-way interaction: feeding practices 3 × age; *P* = 0.002. ^*c*^Two-way interaction: feeding practices 3 × age; *P* = 0.017

**Fig. 2:**
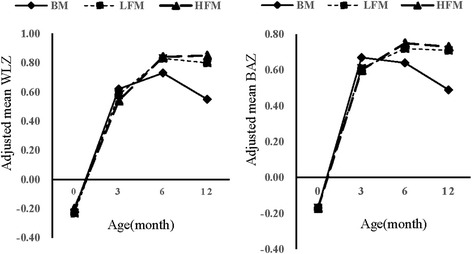
Feeding practice and infant growth. Adjusted mean WLZ and BAZ of infants from birth to 12 mo of age for BM, LFM, and HFM groups. Analyses were performed with the use of linear mixed-effect modeling and adjusted for infant sex, infant birth weight, cesarean, pre-pregnancy BMI, weight gain during pregnancy and WLZ or BAZ at birth. BM: breast milk; LFM: <840 ml formula milk/d; HFM:≧840 ml formula milk/d; WLZ, weight-for-length z score. BAZ, BMI-for-age z scores

In the first 3 mo of life, infants in both the LFM and HFM groups showed similar changes in WLZ and BAZ compared to their BM counterparts (*P* > 0.0.5). From 3 to 6 mo of age, LFM infants showed higher WLZ gain (+0.26 vs +0.12, *P* = 0.016) and BAZ gain (+0.12 vs −0.01, *P* = 0.013) than BM infants. Furthermore, HFM infants showed higher WLZ gain (+0.30 vs +0.12,*P* = 0.023) and BAZ gain (+0.15 vs −0.01, *P* = 0.033) than BM infants. From 6 to 12 mo of age, WLZ and BAZ changes were stable in the LFM and HFM groups, whereas the BM group had less WLZ and BAZ gains than both formula groups; however, the difference was only statistically significant in WLZ change (Fig. 2 and Table 3).

### Logistic regression model results

Table 4 presents the crude and adjusted models for the association between volume of formula milk consumption and the OR of infant greater body weight and overweight at 6 and 12 months. In both crude and adjusted models, lower-volume formula milk feeding (LFM) was not associated with greater body weight and overweight. Higher-volume formula milk feeding (HFM), by contrast, was related to greater body weight and overweight at the ages of 6 and 12 mo. Infants who were HFM fed had 1.60-fold (95% CI 1.05–2.44, *P* = 0.021) higher odds of greater body weight than those who were BM fed at the age of 6 mo, and they had 1.55-fold (95% CI 1.01–2.37, *P* = 0.046) higher odds of greater body weight and 2.13-fold (95% CI 1.03–4.38, *P* = 0.045) higher odds of overweight than BM fed infants at the age of 12 mo.

**Table 4:** Logistic regression analyses for the association between volume of formula milk consumption and the odds of infant’ greater-weight and overweight at the age of 6 and 12 mo

		Greater body weight					Overweight				
			Crude analysis		Adjusted analysis^a^			Crude analysis		Adjusted analysis^a^	
Feeding practices	n (%)	n (%)	OR (CI 95%)	*P* values	OR (CI 95%)	*P* values	n (%)	OR (CI 95%)	*P* values	OR (CI 95%)	*P* values
6mo											
BM	610(55.5)	168(27.5)	Ref.		Ref.		65(10.7)	Ref.		Ref.	
LFM	363(33.2)	115(31.7)	1.22(0.92; 1.62)	0.169	1.25(0.93;1.67)	0.082	35(9.6)	0.90(0.58;1.38)	0.615	0.87(0.56;1.37)	0.551
HFM	120(11.0)	46(38.3)	1.64(1.09; 2.46)*	0.018	1.60(1.05;2.44)*	0.021	15(12.5)	1.20(0.66;2.18)	0.555	1.06(0.56;1.99)	0.880
12mo											
BM	610(55.5)	159(26.1)	Ref.		Ref.		34(5.6)	Ref.		Ref.	
LFM	363(33.2)	112(30.9)	1.27(0.95;1.69)	0.107	1.24(0.92; 1.66)	0.234	27(7.4)	1.36(0.81; 2.30)	0.248	1.47(0.85;2.58)	0.286
HFM	120(11.0)	44(36.7)	1.64(1.09;2.48)*	0.019	1.55(1.01;2.37)*	0.046	12(10.0)	1.88(0.95; 3.75)	0.072	2.13(1.03;4.38)*	0.045

^a^Wald test for heterogeneity and adjusted for infant sex, infant birth WLZ, infant birth weight, introduction of solid foods, cesarean, illness episodes, maternal body mass index pre-pregnancy and weight gain during pregnancy.*p < 0.05. BM, no formula milk; LFM, <840 ml formula milk/d; HFM, ≥840 ml formula milk/d; WLZ, weight-for-length z-score; BAZ, BMI-for-age z score

## Discussion

This study examined the effects of the type and volume of milk fed in early infancy on growth and risk of overweight in later infancy. The results showed that feeding of higher-volumes of formula milk (≥840 ml/d) at 3 month of age was associated with greater body weight and higher risk of overweight at 6 and 12 months of age than was breast milk feeding. The feeding of lower-volume formula milk (<840 ml/d) contributed to higher body weight, body length, and more change in WLZ and BAZ from 3 to 6 month, but had no effect on the risk of overweight in later infancy. These findings were consistent with previous studies that demonstrated that growth in infancy may be affected by both the type and volume of milk fed [10, 14, 15, 22].

Rapid growth in early infancy had been shown to increase the risk of overweight or obesity during later life [2], which conformed to our findings that higher-volume formula feeding contributed to greater body weight and body length, and more change in WLZ and BAZ from 3 to 6 months of age, thus increased risk of overweight in later infancy. A possible explanation for this relationship is that formula-fed infants are always overfed. Firstly, formula-fed infants are fed with bottles [23–25], while breast-fed infants at the 3 mo of age are usually fed directly from the breast because the Chinese mothers has not returned to work according to the China’s labor law. These bottle-fed infants lose their ability to self-regulate intake and delay the satiety response compared with the breast-fed infants [26–28]. Generally, the average volume of infant formula consumed is substantially higher than the volume of breast milk on all days analyzed (about 840 ml/d recommended by the manufacturer for the fully formula-fed infants at 3 mo of age vs 750 ml/d for exclusively breast-fed infants) [29]. The energy content was not determined in the present study, but previous studies reported that the energy content of conventional formula milk (67 kcal/100 ml) was higher than breast milk (65 kcal/100 ml) [13]. Thus, the formula-fed infants consume a higher volume and more energy dense milk and consequently gain more weight.

Another possible explanation is related to “the early protein hypothesis”, which postulate that differences in protein supply between human milk and infant formula play an important role in early programming. This hypothesis assume that more protein intake is causative for a more rapid weight gain in the first 2 y of life and higher risk of obesity observed in formula-fed than in breastfed children [30, 31]. The more rapid weight gain in formula-fed infants might be mediated through an amino acids-induced secretion of insulin and insulin-like growth factor I (IGF-I) [32, 33]. Although the protein intake was not determined in the present study due to lack of data on the volume of breast milk, infants fed with higher volumes formula milk were speculated to consume more protein than breast-fed infants because the protein content was higher in formula milk (1.3~1.7 g/100 ml) than in breast milk (1.0 g~1.3 g/100 ml) [13, 34]. Several other biological mechanisms have been proposed to explain the association between formula feeding and risk of obesity. These include the early differences in the development of gut microbiota and leptin which is found in breast milk but not in formula milk [35–38], or other feeding behaviors [39].

To the best of our knowledge, this is the first study to examine infant growth in lower-and higher-volume formula-fed infants compared to breast-fed infants in Chinese population. The most important strength of this study was that a prospective population-based design were applied to assess growth trajectories and weight status, which helped reduce the risk of recall bias to a minimum. Additional strength of our analysis was its strong design to consider possible residual confounding factors including gestational weight gain, pre-pregnancy BMI, mode of delivery and other feeding or health characteristics of infants, such as records of milk intake, frequency sleep duration, disease condition and vitamin D intake. This allowed us to diminish potential confounders as possible as we can.

Despite the strong design, some infants were unable to be included in the current study because of incomplete data, which may raise the selection bias. Another limitation of this study was that follow-up time of the study population was short and the observed associations may change with increasing follow-up time. Long-term follow-up should be included in the further study to determine whether higher-volume formula intake influence the grow and development and risk of obesity in childhood. In addition, some important factors, such as volume of breastmilk consumption, energy intake, protein intake or detailed records of complementary foods, were not available and analysed in the present study. Finally, WLZ and BAZ were evaluated based on the 2006 WHO growth standards which might be invalid or even misleading for determining the infants’ weight status because the population sample was exclusively breastfed infants from countries other than China.

## Conclusions

In this current study, we found that infants who consumed higher-volumes of formula milk at the age of 3 mo gained more body weight and length in later infancy than breastfed infants. Infants fed with higher-volumes of formula milk seemed to have an increased risk of greater body weight and overweight. Thus, the higher-volume formula feeding should be avoided in the early infancy to prevent overweight or obesity in later infancy. Further studies with more details on milk intake, the energy intake and nutrients intake, are needed to explore the mechanisms behind the association between the higher-volumes of formula consumption and the greater risk of overweight.

## Abbreviations

BAZ: BMI-for-age Z score
BM: No formula milk
BMI: Body Mass Index
HFM: ≥ 840 ml formula milk/d
IGF-I: Insulin-like growth factors-1
LAZ: Length-for-age Z score
LFM: < 840 ml formula milk/d
TMCHC: Tongji Maternal and Child Health Cohort
WAZ: Weight-for-age Z score
WHO: World Health Organization
WLZ: Weight-for-Length Z score
